# Site-specific growth of oriented ZnO nanocrystal arrays

**DOI:** 10.3762/bjnano.10.26

**Published:** 2019-01-24

**Authors:** Rekha Bai, Dinesh K Pandya, Sujeet Chaudhary, Veer Dhaka, Vladislav Khayrudinov, Jori Lemettinen, Christoffer Kauppinen, Harri Lipsanen

**Affiliations:** 1 Thin Film Laboratory, Physics Department, Indian Institute of Technology Delhi, New Delhi 110016, India; 2 Department of Electronics and Nanoengineering, Micronova, Aalto University, Tietotie 3, 02150 Espoo, Finland

**Keywords:** electrodeposition, growth kinetics, nanocrystals, nucleation, twinning, zinc oxide

## Abstract

We report on the growth of ZnO nanocrystals having a hexagonal, prismatic shape, sized 700 nm × 600 nm, on bare indium tin oxide (ITO) substrates. The growth is induced by a low ion flux and involves a low-temperature electrodeposition technique. Further, vertically aligned periodic nanocrystal (NC) growth is engineered at predefined positions on polymer-coated ITO substrates patterned with ordered pores. The vertical alignment of ZnO NCs along the *c*-axis is achieved via ion-by-ion nucleation-controlled growth for patterned pores of size ≈600 nm; however, many-coupled branched NCs with hexagonal shape are formed when a patterned pore size of ≈200 nm is used. X-ray diffraction data is in agreement with the observed morphology. A mechanism is proposed to interpret the observed site-specific oriented/branched growth that is correlated to the pore size. As ordered NC arrays have the potential to generate new collective properties different from single NCs, our first demonstration of a cost effective and facile fabrication process opens up new possibilities for devices with versatile functionalities.

## Introduction

Metal oxide semiconductor nanostructures are quite interesting not only in terms of the basic growth mechanism involved in their fabrication, but also due to the large number of applications based on them in the field of nanoscale optoelectronics [1–4]. ZnO is an important direct band gap (≈3.3 eV), nontoxic, metal oxide semiconductor, which can readily be used for optoelectronic applications. The properties of ZnO can be tailored by changing the morphology of the structures. Thus, fabrication of ZnO having different morphological structures such as nanorods [5–7], nanowires [8], tetrapods [9], nanodisks [10], nanotubes [11], flowers [12], and nanocrystals [13], have been reported. Among the many nanostructured morphologies possible for ZnO, self-assembled ZnO nanocrystals (NCs) have been attracting great attention due to their versatile applications [14]. Self-assembled NC arrays collectively can possibly demonstrate new properties, unlike the fixed properties of single NCs, forming new nanostructures with useful functionalities. Semiconductor NC self-assembly depends on the shape and size of the NCs, a broad range of interactions comprising the cohesive forces of the bulk material, as well as strong coulomb interactions, weak van der Waal forces, and hydrogen bonding. There are reports on the growth of self-assembled twinned pyramids and twinned ZnO nanocrystals [14–16]. The growth of self-assembled NCs is generally reported by solution-based methods such as hydrothermal and solvothermal techniques, which are time consuming, involve multistep methodologies and may be cost ineffective processes [15–20]. In addition to that, these solution-based methods employ a high concentration of precursors and the growth is controlled by some additional reactants during the chemical reaction such as KOH, LiOH and NaOH [21–22]. These additional reactants are employed to reduce the growth rate as well as growth temperature. But, even for a very small concentration of additional reactant added in the reaction bath, the incorporation of some exotic metal ions in the ZnO lattice may produce some inadvertent defect levels and charge carrier recombination centers, in turn deteriorating some of the important material properties. Moreover, these reactants are responsible for changing the surface energy of the crystal facets in an undefined and complex way, which results in the formation of branched nanostructures [21]. Another disadvantage of these solution-based techniques is that the growth takes place in the solution itself and the grown nanocrystals are distributed randomly when collected on the substrate. However, in applications like solar cells based on core/shell ZnO nanocrystals, site-specific growth of the well-aligned nanocrystals is quite important. Therefore, the position-controlled oriented growth of ZnO NC array architectures is highly desirable for practical applications.

In order to simultaneously accomplish the controllable growth of highly ordered as well as highly oriented ZnO NCs with high throughput and maintain low cost for possible large-scale production, we have explored the feasibility of the process based on the combination of employing the use of patterned substrates and a cost-effective growth technique. In particular, we demonstrate the growth of hexagonal faceted self-assembled twin ZnO NCs on bare indium tin oxide (ITO) substrate via a facile low temperature electrodeposition technique that has the potential of yielding good crystal quality with a variety of possible nanoarchitectures under low ion-flux conditions. This growth method has many advantages over the techniques employed in the earlier reports, such as controlled, fast and mass-production process of material fabrication; no requirement of additional reactant (generally used for decreasing the growth temperature and growth rate); cost-effective (due to employing a simple electrolytic bath cell and current source) and ability to grow various nanostructures at ambient pressure and temperature. Moreover, the directed nanocrystal growth can be accomplished on a substrate, rather than in the solution. Furthermore, from the current reported work it is quite difficult to conclude about the nature of twinning, that is, whether twinning appears by joining two NCs or if a single nanocrystal gives rise to the twinned crystal. However, in the electrodeposition technique, since the growth proceeds from a nucleus formed on the conducting substrate used as an electrode, this technique can play an important role to shed some light on the plausible growth mechanism involved in the fabrication of twinned ZnO NCs.

We demonstrate the growth of self-assembled twin ZnO NC arrays at predefined positions by employing polymer-coated ITO substrates patterned with periodic ordered pores. The growth of *c*-axis-aligned twin ZnO nanocrystal arrays was achieved at specific sites via a low-temperature electrodeposition technique with excellent control over orientation, dimension, and location. The effect of a patterned pore size in controlling the growth of array vs branched ZnO NCs is shown. A mechanism based on the nucleation and growth is proposed to understand the oriented/branched twin ZnO NC morphologies.

## Experimental

### Growth of ZnO nanocrystals

**Figure 1:**
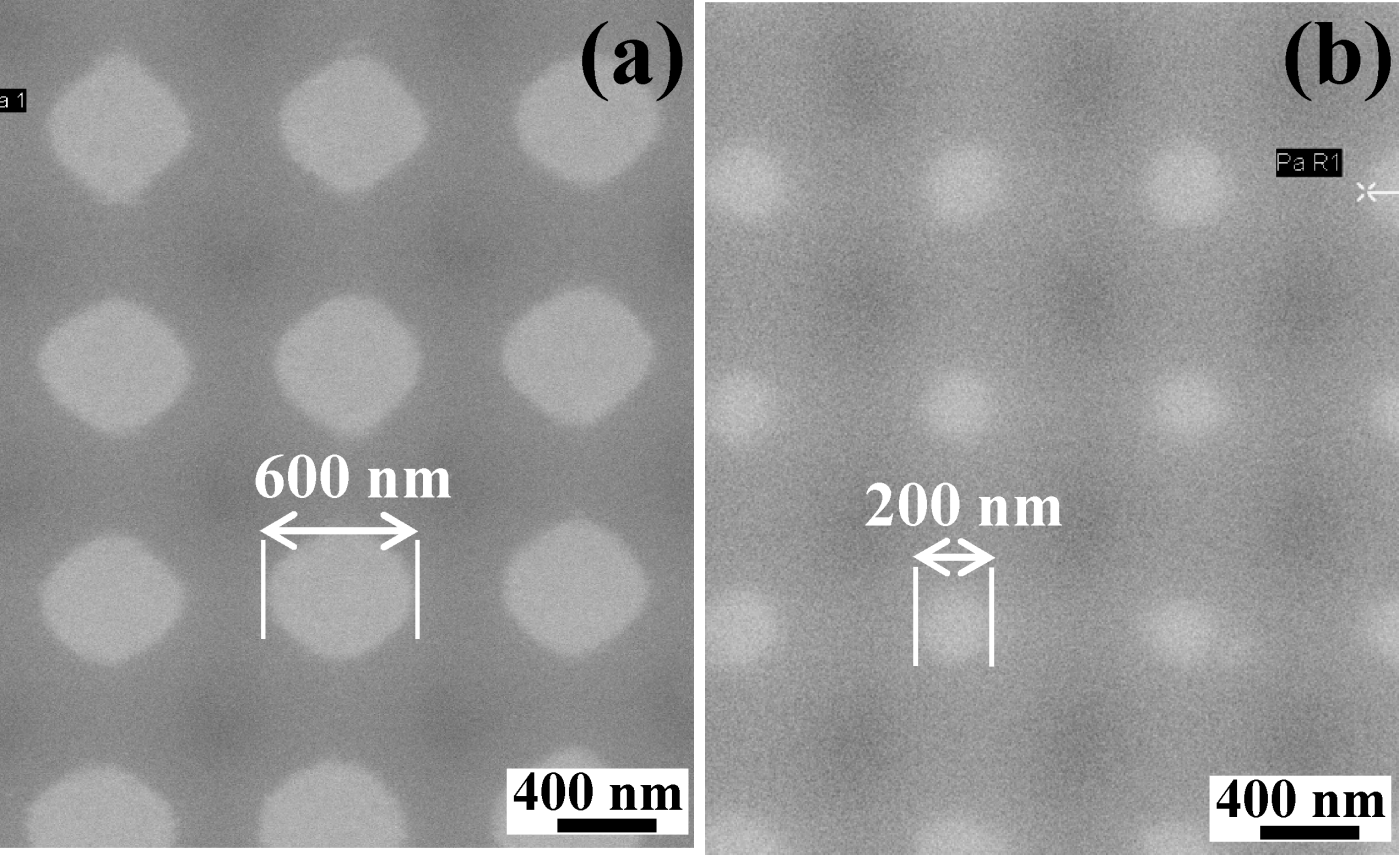
Field emission scanning electron microscopy images of (a) polymer-coated ITO patterned with a pore size of ≈600 nm, and (b) polymer-coated ITO patterned with a pore size of ≈200 nm.

An electrodeposition technique was employed to grow ZnO nanocrystals on both bare and on an array of pores patterned on the polymer-coated indium-doped tin oxide (ITO) conducting substrates. The patterning process for the polymer, poly(Disperse Red 1 acrylate), involves laser interference lithography and oxygen plasma etching and has been reported in detail previously [23]. Two different sizes of pores with diameter ≈600 and ≈200 nm patterned on the ITO substrate employed in the present work are shown in Figure 1. The period of the pores was kept nearly the same. The electrodeposition process was carried out in a specially designed, closed, three electrode, glass cell. The bare/polymer-coated patterned ITO substrates were used as a working electrode (2 × 2 cm^2^) while a platinum sheet (2.5 × 2.5 cm^2^) and a saturated calomel electrode (SCE) were used as the counter and reference electrodes, respectively. The electrolyte (bath) temperature was kept at 60 °C. For the growth of ZnO, the precursor solution was obtained from the 1 mM Zn(NO_3_)_3_·6H_2_O. The electrochemical deposition of ZnO was carried out in solution having pH ≈5.6 at deposition potential −1.0 V (vs SCE) for 15 minutes using an electrochemical analyzer (CHI1104A). After deposition, the sample was removed from the electrolyte and rinsed in deionized water [24]. The sample grown on bare ITO is named as SB; the samples grown on patterned ITO with pore size ≈600 and ≈200 nm are named as S600 and S200, respectively.

### Characterization of ZnO nanocrystals

The ZnO nanocrystals grown on bare/patterned ITO were structurally characterized using an X-ray diffractometer (PANalytical X’pert Pro model) having Cu Kα radiation (λ = 1.54 Å) in the 2θ range 20° to 80° using grazing incidence X-ray diffraction (GIXRD). The setup consists of an X-ray mirror, a Ni filter, and a PIXcel3D detector in scanning line mode. The surface morphology of the prepared samples was investigated by using a field emission scanning electron microscopy (FESEM) using a Zeiss Supra 40 device. A JEOL 2200FS transmission electron microscope (TEM) was used to investigate the crystallinity of the NCs.

## Results and Discussion

**Figure 2:**
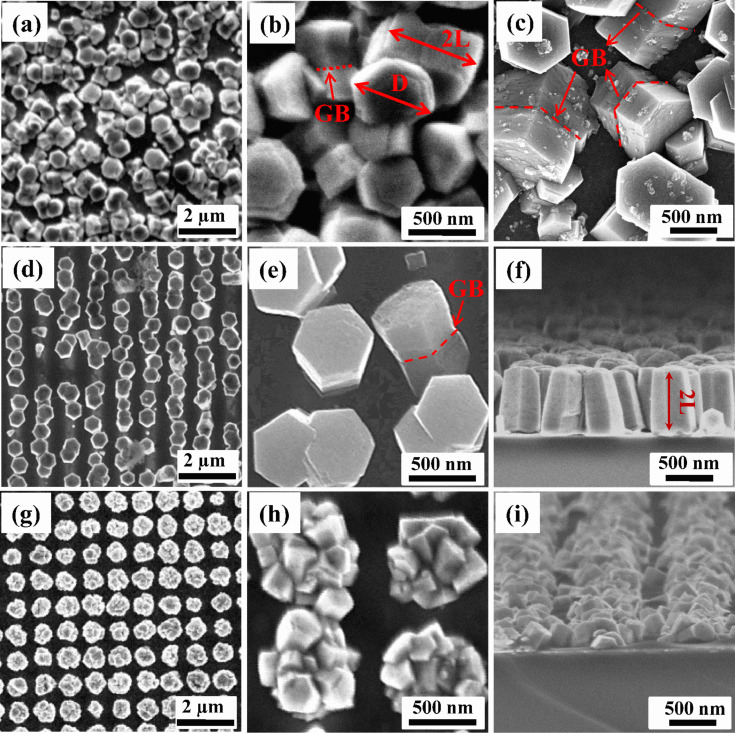
Top view FESEM images of ZnO NCs for (a, b, c) SB (on bare ITO) (d, e) S600 (on patterned ITO with pore size ≈600 nm), (g, h) S200 (on patterned ITO with pore size ≈200 nm) at low magnification and at high magnification; and the cross-sectional view for (f) S600 (i) S200, respectively.

The FESEM images of ZnO NCs grown on bare ITO and on the array of pores patterned on the polymer-coated ITO substrates are shown in Figure 2. ZnO NCs grown on bare ITO are shown in Figure 2a–c and exhibit hexagonal prismatic shape that is characteristic of the wurtzite structure of ZnO. The crystals are well separated from each other and are oriented randomly on the substrate. This exhibits a central grain boundary (GB) perpendicular to the elongation direction (marked with dotted line in Figure 2b,c) that is generally assigned to twinning. These crystals with well-defined hexagonal face and side facets possess an overall length (2L) of ≈700 nm and a width/diagonal (D) of ≈600 nm. Typically, the top hexagonal faces of nanocrystals are composed of flat hexagonal terraces as seen in Figure 2b,c, characteristic of the layer-by-layer growth mechanism and are thus a clear indication of the ion-by-ion nucleation-controlled deposition process [5,25]. So, we observe that twinned ZnO NCs with almost the same diagonal dimension grow on bare ITO, though in random orientations. However, these become vertically aligned when grown in pores patterned on an ITO substrate with pore diameter ≈600 nm. Figure 2d–f shows the growth of such ZnO NCs (sample S600). It can be seen that the ZnO NCs grow in the pores on the ITO surface which is not covered with polymer. The growth of NCs is quite periodic as guided by the pore pattern. It can be further seen that the twinned ZnO NCs are grown in such a way that hexagonal faces, that define the *c*-axis, are parallel to the substrate. In Figure 2e,f, the central grain boundary perpendicular to the elongation direction can be clearly seen and indicates that polymer-assisted growth on ITO did not affect the morphology of twin NCs but helps to align them with their *c*-axis normal to the substrate. Moreover, the length and width of crystals are same as observed in the SB sample. Thus, we can say that the periodic array of *c*-axis-oriented twinned NCs at predetermined sites can be grown by patterning the polymer-coated ITO substrate. We also tried to control the width of well-aligned ZnO NCs by employing ITO patterned with a pore size of ≈200 nm (Figure 2g–i). Surprisingly, in contrast to the S600 sample, wherein crystals with *c*-axis normal to the ITO substrate are formed, many coupled branched ZnO NCs with hexagonal shape are revealed when the pore size was decreased to ≈200 nm (sample S200), demonstrating the significant effect of pore size on the morphology of ZnO NCs. However, it may be pointed out that although the overall morphology of the NCs is changed in S200, the periodicity observed is maintained in both S600 and S200 samples as per the pore pattern planned on the ITO substrate.

Now, the growth of twinned NCs on ITO substrate can be understood in terms of nucleation and growth kinetics involved during the fabrication process. The electrodeposition technique employed in this work rules out the formation of twinned ZnO NCs by co-joining two individual crystals of length ≈*L* (as is possible in solution-based techniques where two crystals independently formed by homogeneous nucleation in the solution itself can get coupled) as the growth starts by heterogeneous nucleation on the ITO surface. Since twinned crystals are observed to be formed, it is plausible that initially ZnO NCs having length ≈*L* and width ≈*D* grow on the bare ITO surface, and subsequently, a second set of crystalline material starts to grow on the top of first crystal and, in the process, leads to formation of twinned ZnO NCs. It is interesting to note that both the crystals forming the twin are almost of same dimensions, *L* and *D*. The obvious question is what gives rise to twin formation. The twin growth can be understood as follows. ZnO crystal structure consists of hexagonally close packed oxygen and zinc atoms. ZnO crystals consists of a top tetrahedron corner-exposed polar zinc (0001) face, six symmetric non-polar {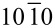} planes parallel to the [0001] direction, and a basal polar oxygen (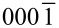) face [17]. It is well known that the hexagonal wurtzite ZnO has two polar planes (0001) and (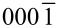), which have high surface energy that can absorb new small particles to reduce its surface energy and thus ZnO NCs are oriented to grow along the [0001] direction [17]. The attractive force between the two basal planes is a prime requirement to make a twinned crystal. So, two negatively charged O (or positively charged Zn-terminated) crystal planes of ZnO can be linked together by adsorption of positively (or negatively) charged species. In the present work, only Zn(NO_3_)_3_·6H_2_O is used as precursor to fabricate ZnO crystals and the reaction involved for the formation of ZnO crystals is as follows:


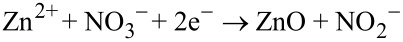


As only NO_2_^−^ ions are released during the reaction, so we can say that the two positively charged Zn-terminated polar planes are connected together by adsorption of NO_2_^−^ and this could understandably account for the attractive force needed between two Zn-terminated basal planes for eventual formation of twinned NCs of ZnO.

The growth of *c*-axis oriented/branched ZnO NCs can be correlated to pore-size-dependent growth kinetics of ZnO crystals. In the case of the S600 sample that exhibits a vertical array of ZnO nanocrystals, the size of the pore was approximately equal to the width of ZnO NCs that are formed on bare ITO (sample SB). The growth conditions, still being same, allow the unhindered growth of ZnO NCs identical to that of the SB case, except only on the specific sites on the ITO surfaces exposed by patterning that are available for the heterogeneous nucleation. As the size of the pore was equal to the width of ZnO NCs grown on bare ITO, the lateral growth on the substrate is not restricted by the walls of the pores; however, the growth direction becomes aligned normal to the substrate. It is well known that ZnO crystals can be grown on ITO substrate with the (0001) plane parallel to the substrate plane. This results in the formation of *c*-axis-oriented NCs and twinning appears as explained earlier. But, in the case of S200 sample, the size of the patterned pore is three times smaller than the width of the crystals grown in case of SB and S600 samples. This results in the constrained lateral growth due to the walls of the pore and results in increased surface energy at the side faces of ZnO NCs. The constrained growth continues until the ZnO crystal grows to fill the pore and comes above the pore walls. Subsequently the seven surfaces (six sides and one top surface) serve as secondary nucleation sites and growth restarts from these high surface energy sites in order to reduce the surface energy accumulated from pore wall constrained growth. The branched coupled crystals are thus formed. So, we can conclude that the size of the patterned pore plays a crucial role to determine the morphology developed during the growth process.

**Figure 3:**
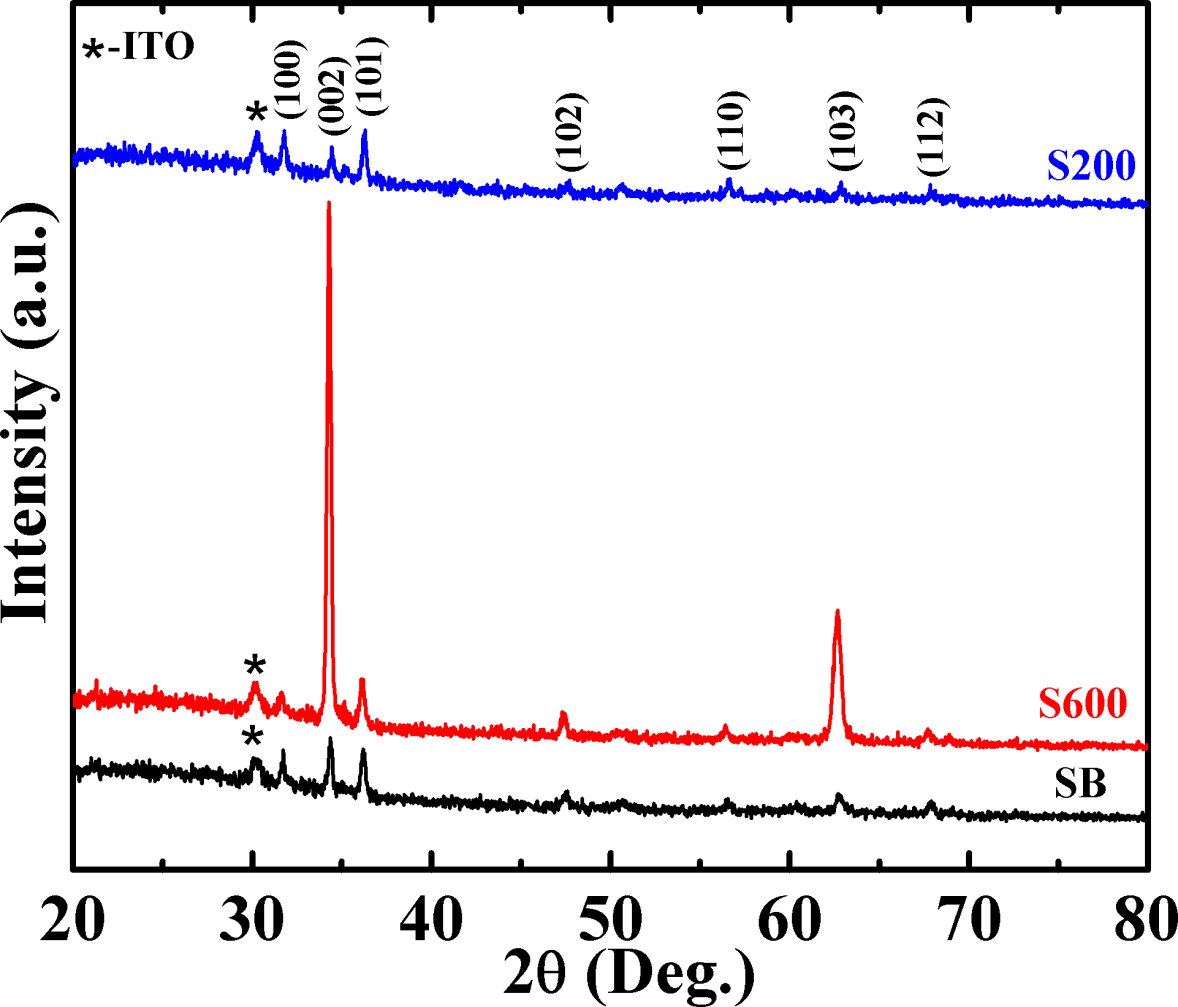
X-ray diffraction spectra of ZnO NCs for SB (on bare ITO), S600 (on patterned ITO with pore size ≈600 nm), and S200 (on patterned ITO with pore size ≈200 nm).

XRD spectra of twinned ZnO NCs grown on the bare and patterned ITO substrates are shown in Figure 3. The XRD patterns of all the samples show that all the observed peaks correspond to hexagonal wurtzite phase (JCPDS 05-0664) of ZnO. The absence of any additional peak suggests that no other phase is formed. The peak marked by (*) emerged from the underlying ITO substrate. The substrate peak has relatively low intensity since the used GIXRD setup measures the volume close to the sample surface. It can be further seen that the XRD pattern of ZnO NCs grown on bare ITO does not show any preferential orientation for a particular plane and indicates the random orientation of the crystals grown on the bare ITO substrate, which is in accordance with the FESEM observations (Figure 2a). In contrast, the XRD pattern of S600 is dominated by a sharp diffraction peak at 34.4° corresponding to (002) planes and is indicative of the growth of ZnO NCs with the *c*-axis perpendicular to the substrate [5]. Some weak peaks corresponding to ZnO phase, e.g., (103), are also present on account of the slight tilt of the NCs away from vertical. The XRD results and FESEM images (Figure 2d) exhibiting vertically standing hexagonal prisms are consistent with each other. However, the XRD spectra of S200 shows no preferential orientation of any particular plane. This is in accordance with the FESEM data of the sample (Figure 2g), where ZnO NCs with branches oriented in various non-vertical directions are observed. Thus, we can conclude that the observed FESEM and XRD observations are consistent with each other with respect to the oriented and aligned growth of ZnO NCs or otherwise.

**Figure 4:**
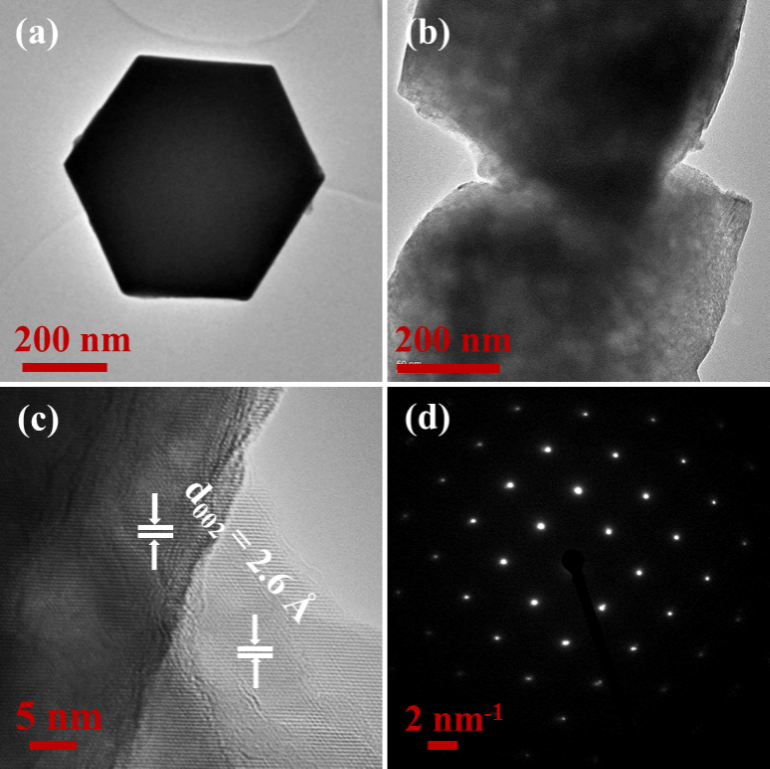
Microstructure characterization of hexagonal-shaped twinned ZnO NCs for the S600 sample. (a, b) Low-resolution TEM image, (c) high-resolution TEM image, and (d) selected area electron diffraction pattern.

To further examine the crystal structure and morphology of the twinned ZnO NCs, TEM measurements were performed and the recorded images for the S600 sample are shown in Figure 4. Figure 4a,b shows the low-resolution bright-field TEM images of the hexagonal-shaped twinned S600 ZnO NCs. The figures reveal a smooth and clean surface with flat hexagonal terraces at the top of ZnO NCs having width/diagonal *D* ≈ 600 nm, which is consistent with the observed FESEM in Figure 2d,e. The corresponding high-resolution TEM (HRTEM) image is recorded to further investigate the morphological characteristics of ZnO NCs. The observed sharp lattice fringes in the HRTEM image reveal the good crystallinity of twinned ZnO NCs. This is possibly due to the low ion flux arriving at the substrate surface that promotes the formation of well-ordered hexagonal-shaped twinned ZnO NCs followed by a controlled heterogeneous ion-by-ion growth mechanism [5–6] as demonstrated by the lattice fringes in Figure 4c. The NCs exhibit interplanar spacing of *d* ≈ 2.6 Å belonging to the (002) lattice plane of the wurtzite phase of ZnO (JCPDS 05-0664), which is well in agreement with the XRD results (Figure 3). Moreover, the selected area electron diffraction (SAED) pattern (Figure 4d) reveals the excellent crystallinity of ZnO NCs and confirms the growth of the *c*-axis normal to the substrate in these twinned ZnO NCs.

Now we compare our growth method and quality of twinned ZnO NCs with similar structures reported previously. Greer et al. [15] have employed gelatin as the structure-directing agent to fabricate twinned ZnO NCs. The removal of embedded gelatin in NCs requires calcination at 600 °C, which may in turn impact the physical properties on account of the known role of oxygen vacancies and defects on electron transport behavior of ZnO [26–27]. But in our case no such high temperature processing is required at any stage. Cho et al. [17] have used a solution method with tri-potassium citrate and trisodium citrate as additional reagents to grow the twinned ZnO NCs. The growth method they adopted involves multiple steps and is quite time consuming as zinc acetate dihydrate and ammonia solution containing citrates is initially kept at 90 °C for one hour in a Teflon-lined autoclave and subsequently dried in an oven at 60 °C for 12 hours. Taubert et al. [18–20] have employed different polymers to grow the twinned ZnO NCs, but the surface quality and flatness of their NCs are quite low compared to those synthesized in our case, which are almost atomically flat. In addition to that, none of the previous reports exhibit fabrication of well-aligned periodic arrays of ZnO NCs and site-specific growth, which is quite essential for their use in novel technological applications.

## Conclusion

The growth of twinned nanocrystals of ZnO, 700 nm length and 600 nm width, with hexagonal prismatic shape has been demonstrated by employing a low-temperature single-step electrodeposition process, free of any supplementary reactants/additives, by creating low ion-flux conditions. It is further demonstrated that the ITO substrate patterned with a pore size of ≈600 nm provides site-specific growth centers for fabrication of ordered arrays of twin ZnO NCs with their *c*-axis [0001] perpendicular to the substrate plane. In contrast, a substrate patterned with a pore size of ≈200 nm (significantly smaller than the width of the NCs that form under uninhibited growth on the bare ITO surface) leads to the formation of coupled branched ZnO NCs. Plausible growth mechanisms underlying the formation of twinned crystals oriented/branched ZnO NCs are presented. The formation of twins seems to be facilitated by the linking of the two positively charged Zn polar surfaces of ZnO NCs by the negatively charged NO_2_^−^ ions. The formation of branched structures is attributed to the constrained lateral growth due to limit imposed by the pore walls and the associated increase in surface energy. In the absence of such a constraint, for example, in the case where the pore size matches the crystal width, a vertically aligned twinned NC array is formed. Such a simple, cost effective and large area, scalable fabrication of ZnO NC array potentially opens a path for new device possibilities with novel functionalities.
